# Halogenated Terpenes and a C_15_-Acetogenin from the Marine Red Alga *Laurencia saitoi*

**DOI:** 10.3390/molecules13112894

**Published:** 2008-11-20

**Authors:** Nai-Yun Ji, Xiao-Ming Li, Bin-Gui Wang

**Affiliations:** 1 Yantai Institute of Coastal Zone Research for Sustainable Development, Chinese Academy of Sciences, Yantai 264003, P. R. China; 2 Institute of Oceanology, Chinese Academy of Sciences, Qingdao 266071, P. R. China; E-mails: lixmqd@yahoo.com.cn (X-M. L.); wangbg@ms.qdio.ac.cn (B-G.W.)

**Keywords:** *Laurencia saitoi*, Diterpene, Triterpene, C_15_-acetogenin

## Abstract

Seven parguerane diterpenes: 15-bromo-2,7,19-triacetoxyparguer-9(11)-en-16-ol (**1**), 15-bromo-2,7,16,19-tetraacetoxyparguer-9(11)-ene (**2**), 15-bromo-2,19-diacetoxy-parguer-9(11)-en-7,16-diol (**3**), 15-bromo-2,16,19-triacetoxyparguer-9(11)-en-7-ol (**4**), 15-bromo-2,16-diacetoxyparguer-9(11)-en-7-ol (**5**), 15-bromoparguer-9(11)-en-16-ol (**6**), 15-bromoparguer-7-en-16-ol (**7**), two polyether triterpenes: thyrsiferol (**8**) and thyrsiferyl 23-acetate (**9**), and one C_15_-acetogenin, neolaurallene (**10**), were isolated from a sample of marine red alga *Laurencia saitoi* collected off the coast of Yantai. Their structures were established by detailed NMR spectroscopic analysis and comparison with literature data.

## Introduction

**Figure 1:**
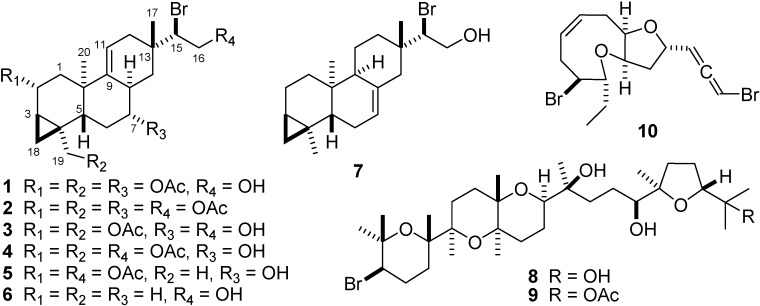
Structures of compounds **1**-**10**.

The marine red algae of the genus *Laurencia* (family Rhodomelaceae, order Ceramiales) comprise about 135 species worldwide, which are mainly spread along tropical, subtropical, and temperate coasts [1]. The taxonomy of *Laurencia* species has been widely studied and is often confusing due to the high morphological variability within individual species. The secondary metabolites of the genus *Laurencia*, which have been mostly investigated since the 1960s, are mainly composed of sesquiterpenes, diterpenes, triterpenes, and C_15_-acetogenins [2], and they are characteristic of individual *Laurencia* species [1,3]. Our chemical investigation of the marine red alga *L. saitoi* collected off the coast of Yantai revealed a new naturally occurring diterpene, 15-bromo-2,7,19-tri-acetoxyparguer-9(11)-en-16-ol (**1**) [4], together with nine known natural products: 15-bromo-2,7,16,19-tetraacetoxyparguer-9(11)-ene (**2**) [5], 15-bromo-2,19-diacetoxyparguer-9(11)-en-7,16-diol (**3**) [4], 15-bromo-2,16,19-triacetoxyparguer-9(11)-en-7-ol (**4**) [4], 15-bromo-2,16-diacetoxyparguer-9(11)-en-7-ol (**5**) [6], 15-bromoparguer-9(11)-en-16-ol (**6**) [7], 15-bromoparguer-7-en-16-ol (**7**) [7], thyrsiferol (**8**) [8], thyrsiferyl 23-acetate (**9**) [9], and neolaurallene (**10**) [10,11]. The isolation and structural determination of compounds **1**-**10** (Figure 1) are the subject of this paper.

## Results and Discussion

The dried and powdered alga *L. saitoi* was extracted with the mixture of CHCl_3_ and MeOH (2:1, v/v). The concentrated extracts were partitioned between H_2_O and EtOAc. The EtOAc-soluble fraction was purified by a combination of silica gel and Sephadex LH-20 column chromatography, as well as preparative TLC procedures, to yield compounds **1**-**10**.

Compounds **1**-**4** exhibited similar ^1^H-NMR spectra, which all displayed two upfield methyls, two oxygenated methylenes, three oxygenated (or halogenated) methines, and one olefinic proton. Additionally, three, four, two, and three acetyl group methyls were observed in compounds **1**-**4**, respectively. Thus, **1**-**4** differed from each other in the number and position of acetyl groups. The structures of **1**-**4** were preliminarily assigned by comparison of their ^1^H-NMR data with those in literature [4,5], and the structure of **4** was unambiguously established by comparison of its ^13^C-NMR and DEPT spectroscopic data with those in the literature [4]. A detailed comparison of the ^13^C-NMR and DEPT spectroscopic data of **3** with those of **4** confirmed that **3** was a deacetylated derivative of **4** at C-16 by the lack of one acetyl group and 9.5 ppm downfield shift of C-15 and 1.7 ppm upfield shift of C-16 in the ^13^C-NMR spectrum of **3**[4,12]. Compound **2** was established as a C-7 acetylated derivative of **4**, as indicated by the presence of an additional acetyl group and a 1.4 ppm downfield shift of C-7 in the ^13^C-NMR spectrum [4,12].

**Table 1:** ^13^C-NMR data of compounds **1**-**10** (125 MHz, in CDCl_3_).

	1	2	3	4	5	6	7	8	9	10
1	37.4 t	37.4 t	37.5 t	37.4 t	38.2 t	31.1 t	30.3 t	31.0 q	31.0 q	73.9 d
2	68.2 d	68.2 d	68.3 d	68.3 d	69.4 d	19.4 t	19.2 t	75.0 s	75.0 s	201.4 s
3	22.0 d	21.9 d	22.1 d	22.1 d	23.6 d	19.3 d	20.6 d	59.0 d	58.9 d	102.2 d
4	20.7 s	20.6 s	20.8 s	20.8 s	17.2 s	16.3 s	15.1 s	28.3 t	28.2 t	74.5 d
5	45.6 d	45.6 d	45.8 d	45.8 d	46.6 d	50.1 d	38.6 d	37.0 t	37.0 t	39.0 t
6	29.5 t	29.5 t	33.5 t	33.5 t	34.7 t	25.5 t	27.0 t	74.4 s	74.4 s	72.8 d
7	78.2 d	78.3 d	76.7 d	76.9 d	76.7 d	35.9 t	121.1 d	86.6 d	86.5 d	79.7 d
8	35.2 d	35.2 d	38.3 d	38.3 d	38.4 d	30.7 d	136.3 s	23.0 t	23.0 t	26.8 t
9	142.1 s	142.2 s	142.9 s	143.1 s	143.5 s	147.1 s	50.3 d	38.6 t	38.5 t	127.3 d
10	36.5 s	36.4 s	36.5 s	36.5 s	37.1 s	37.5 s	32.4 s	72.0 s	71.9 s	129.3 d
11	118.5 d	118.3 d	117.7 d	117.5 d	117.2 d	114.4 d	24.6 t	76.4 d	76.3 d	34.7 t
12	37.4 t	37.4 t	37.9 t	38.0 t	38.1 t	39.3 t	37.1 t	21.2 t	21.2 t	52.8 d
13	35.2 s	35.3 s	35.3 s	35.4 s	35.5 s	35.6 s	39.8 s	20.7 t	20.7 t	84.4 d
14	38.8 t	38.6 t	38.9 t	38.8 t	38.8 t	41.8 t	46.8 t	76.1 d	76.1 d	23.2 t
15	68.7 d	58.9 d	69.0 d	59.5 d	59.6 d	70.0 d	76.4 d	73.3 s	73.2 s	11.4 q
16	64.4 t	65.9 t	64.3 t	66.0 t	66.1 t	64.5 t	63.8 t	33.6 t	33.6 t	
17	24.7 q	24.2 q	24.8 q	24.3 q	24.3 q	24.9 q	19.4 q	25.5 t	25.4 t	
18	18.9 t	18.8 t	18.8 t	18.8 t	21.7 t	21.4 t	19.7 t	77.7 d	77.5 d	
19	69.6 t	69.5 t	69.8 t	69.8 t	23.2 q	24.1 q	24.6 q	86.1 s	86.3 s	
20	19.9 q	19.6 q	20.0 q	19.8 q	19.6 q	17.9 q	19.5 q	32.5 t	31.9 t	
21								26.6 t	26.8 t	
22								87.5 d	85.8 d	
23								70.5 s	82.5 s	
24								24.0 q	22.1 q	
25								23.7 q	23.7 q	
26								20.1 q	20.1 q	
27								21.4 q	21.4 q	
28								22.9 q	22.9 q	
29								23.4 q	23.2 q	
30								27.6 q	22.4 q	
CH_3_CO	21.6 q	21.5 q	21.6 q	21.6 q	21.6 q				22.0 q	
CH_3_CO	21.2 q	21.2 q	21.1 q	21.1 q	20.9 q					
CH_3_CO	21.1 q	21.1 q		20.9 q						
CH_3_CO		20.9 q								
CH_3_CO	170.9 s	170.8 s	170.9 s	170.9 s	170.7 s				170.4 s	
CH_3_CO	170.6 s	170.6 s	170.6 s	170.7 s	170.6 s					
CH_3_CO	170.5 s	170.6 s		170.5 s						
CH_3_CO		170.4 s								

Further, compound **1** was established as the C-16 deacetylated derivative of **2** based on the disappearance of one acetyl group and 9.8 ppm downfield shift of C-15 and 1.5 ppm upfield shift of C-16 in the ^13^C-NMR spectrum of **1** [4,12]. Compound **1** has been previously reported as a derivative of **2**, obtained after saponification of **2** with aqueous 10% Na_2_CO_3_ at room temperature [4]. The structures of **5**-**10** were unambiguously established by comparison of their ^1^H and ^13^C-NMR data with those in the literature [6,7,8,9,10,11]. The ^13^C-NMR data of compounds **1**-**10** are shown in Table 1, and those of **1**-**3** are reported for the first time.

Previous investigations revealed that the secondary metabolites of *L. saitoi* from the western Pacific Ocean, which was misidentified as *L. obtusa* due to their morphological similarity [13], comprised parguerane diterpenes, polyether triterpenes, and two sesquiterpenes [4,9,14,15,16,17,18]. Parguerane diterpenes which possess a unique modified pimarane skeleton were firstly isolated from the sea hare *Aplysia dactylomela* and have subsequently only been found in several other *Laurencia* species, such as *L. obtusa*, *L. nipponica*, and *L. filiformis* [5,6,7,12]. Our study confirms the view that parguerane diterpenes and polyether triterpenes together are characteristic of the chemical composition of *L. saitoi*. On the other hand, this is the first report of the occurrence of a C_15_-acetogenin in *L. saitoi* and compounds **1**, **6**, and **7** add to the molecular diversity of parguerane diterpenes present in this species.

## Experimental

### General

NMR spectra were recorded at 500 and 125 MHz for ^1^H and ^13^C, respectively, on a Bruker Avance 500 MHz NMR spectrometer in CDCl_3_ with TMS as internal standard. Column chromatography was performed with silica gel (200-300 mesh, Qingdao Haiyang Chemical Co., Qingdao, P.R. China) andSephadex LH-20 (Pharmacia). TLC was carried out with precoated silica gel plates (GF-254, Qingdao Haiyang Chemical Co., Qingdao, P.R. China). All solvents were of analytical grade.

### Algal Material

The red alga *Laurencia saitoi* Perestenko was collected off the coast of Yantai (lat. 37°31’15”N, long. 121°26’59”E), Shandong Province, P. R. China, in July 2008. It was identified by one of the authors (Nai-Yun Ji) and a voucher specimen (MRA0807) has been deposited at the Bio-Resource Laboratory of Yantai Institute of Coastal Zone Research for Sustainable Development, Chinese Academy of Sciences.

### Extraction and Isolation

Dried and powdered alga *L. saitoi* (70 g) was extracted with the mixture of CHCl_3_ and MeOH (2:1, v/v). The concentrated extract was partitioned between H_2_O and EtOAc. The EtOAc-soluble fraction was fractioned by silica gel column chromatography (petroleum ether (PE)/EtOAc gradient) to give six fractions, I-VI. The fraction II, eluted with PE/EtOAc 100:1, was purified by Sephadex LH-20 column chromatography (CHCl_3_/MeOH 1:1) to afford **10** (43.0 mg). The fraction III, eluted with PE/EtOAc 30:1, was purified by Sephadex LH-20 column chromatography (CHCl_3_/MeOH 1:1) and preparative TLC (PE/CHCl_3_ 1:1) to afford **6** (3.8 mg) and **7** (3.8 mg). The fraction VI, eluted with EtOAc, was purified by Sephadex LH-20 column chromatography (CHCl_3_/MeOH 1:1) and preparative TLC (CHCl_3_/EtOAc 4:3) to afford **1** (4.9 mg), **2** (51.6 mg), **3** (10.8 mg), **4** (17.3 mg), **5** (14.5 mg), **8** (8.5 mg), and **9** (31.7 mg).
